# β2-Adrenoceptors and GRK2 as Potential Biomarkers in Patients With Chronic Pulmonary Regurgitation

**DOI:** 10.3389/fphar.2019.00093

**Published:** 2019-02-19

**Authors:** María Rodriguez-Serrano, Joaquín Rueda, Francisco Buendía, Fermi Monto, Jaime Aguero, Ana Osa, Oscar Cano, Luis Martínez-Dolz, Pilar D’Ocon

**Affiliations:** ^1^Servicio de Cardiología, Hospital de Manises, Valencia, Spain; ^2^Servicio de Cardiología, Hospital Universitario y Politécnico La Fe, Valencia, Spain; ^3^Departamento de Farmacología, Facultad de Farmacia, Universitat de València, Valencia, Spain; ^4^Estructura de Recerca Interdisciplinar en Biotecnologia i Biomedicina (ERI BIOTECMED), Universitat de València, Valencia, Spain; ^5^Área de Fisiopatología del Miocardio, Centro Nacional de Investigaciones Cardiovasculares Carlos III (CNIC), Madrid, Spain

**Keywords:** pulmonary regurgitation, pulmonary valve replacement, right ventricle, congenital heart disease, β2-adrenoceptor, GRK2

## Abstract

Pulmonary regurgitation (PR) is a frequent complication after repair of congenital heart disease. Three different GRK isoforms (GRK2, GRK5, and GRK3) and two β-adrenoceptors (β1-AR and β2-AR) are present in peripheral blood mononuclear cells (PBMC) and their expression changes as a consequence of the hemodynamic and neurohumoral alterations that occur in some cardiovascular diseases. Therefore, they could be useful as biomarkers in PR. A prospective study was conducted to describe the expression (TaqMan Gene Expression Assays) of β-ARs and GRKs in PBMC isolated (Ficoll^®^ gradient) from patients with severe PR before and after pulmonary valve replacement and establish if this expression correlates to clinical status. 23 patients with severe PR were included and compared with 22 healthy volunteers (controls). PR patients before the PVR had a significantly lower expression of β2-AR (513.8 ± 261.2 mRNA copies) vs. controls (812.5 ± 497.2 mRNA copies), so as GRK2 expression (503.4 ± 364.9 copies vs. 858.1 ± 380.3 mRNA copies). The expression of β2-AR and GRK2 significantly decreases in symptomatic and asymptomatic patients, as well as in patients under treatment with beta-blockers and non-treated patients. The expression of β2-AR and GRK2 in PR patients recovers the normal values after pulmonary valve replacement (754,8 ± 77,1 and 897,8 ± 87,4 copies, respectively). Therefore, changes in the expression of β2-AR and GRK2 in PBMC of PR patients, could be considered as potential biomarkers to determine clinical decisions.

## Introduction

The β-adrenergic system plays a key role in the regulation of heart function and it is also known that it is involved in the pathogenesis of heart failure (HF). In this pathology, levels of circulating catecholamines increase and this determines an adaptation in the expression and activity of adrenoceptors (AR) as well as the G-protein coupled receptors kinases (GRK), which phosphorylate AR when they are occupied by agonists.

Three different GRK isoforms (GRK2, GRK5 and, in a minor proportion, GRK3) are present in the heart and also in peripheral blood mononuclear cells (PBMC) (Vinge et al., 2007; Grisanti et al., 2018) and their expression (especially GRK2) changes as a consequence of the hemodynamic alterations that occur in HF or hypertension (Iaccarino et al., 2005; Penela et al., 2006; Vinge et al., 2007; Izzo et al., 2008; Cohn et al., 2009; Santulli et al., 2011, 2013; Piao et al., 2012; Grisanti et al., 2018). Moreover, it is remarkable that expression of β2-ARs and GRK2 follow a similar pattern of changes in different tissues and pathologies (Montó et al., 2015). Initially, changes in the expression of GRKs and β-ARs were determined in myocytes (endomyocardial biopsies, cardiac explant), but it is also possible to study these changes in PBMC (Iaccarino et al., 2005; Agüero et al., 2008; Oliver et al., 2010). Thus, their determination in peripheral blood can become a useful parameter for the follow-up of cardiovascular disease. In fact, in the study of HF, the expression of GRK2 in circulating lymphocytes (Rengo et al., 2016) has been used as a molecular marker.

Pulmonary regurgitation (PR) occurs frequently after surgical repair of tetralogy of Fallot and other congenital heart diseases (CHD), which results in a right ventricle (RV) volume overload and progressive dysfunction (Gregg and Foster, 2007). Chronic severe PR is associated with reduced exercise capacity, increased risk of arrhythmias and sudden cardiac death (Bouzas et al., 2005). Pulmonary valve replacement (PVR) is indicated in symptomatic patients or with moderate-severe RV dilatation/dysfunction (Warnes et al., 2008; Baumgartner et al., 2010). It results in improvement of functional class and reduction of RV volumes, but after surgery there is no evidence of arrhythmias reduction, improvement in exercise capacity, RV systolic function or survival (Ferraz Cavalvanti et al., 2013). Therefore, many authors recommend performing PVR earlier. The risk of the surgery is low (Geva, 2013), but the tissue valves deteriorates and multiple re-interventions are necessary (Oliver et al., 2015) then, the time for PVR must be adequately selected to obtain the maximal clinical benefit with the minimum number of re-interventions. In this context, the existence of a molecular marker, as occurs with GRK2 in HF, could be a useful tool to determine the clinical actuation.

In a previous work we have found that the gene expression pattern of GRK2 and the β2-AR was altered in patients with severe PR in a similar way to patients with advanced HF (Rodríguez-Serrano et al., 2018). In the present work, we propose a molecular approach to improve the pathophysiological knowledge of PR in order to help us to decide the right time for PVR. Our main objective was to describe changes in gene expression of β1 and β2 ARs and GRKs (GRK2, GRK3, and GRK5) in PBMC from patients with severe PR, before and after PVR, and establish if this expression correlates to clinical status.

## Materials and Methods

Patients with severe PR followed in the adult congenital heart disease unit from December 2011 to July 2015 were selected prospectively. The diagnosis of severe PR was made following echocardiographic criteria (Lancellotti et al., 2013) and was confirmed by magnetic resonance imaging. Patients with left ventricular dysfunction or other severe valve disease were excluded. After signing the informed consent, a blood sample was taken for analysis of the gene expression of β-ARs (β1 and β2) and GRKs (GRK2, GRK3, and GRK5) in PBMC. Clinical data (functional class of the New York Heart Association, rhythm and width of QRS) and RV assessment by cardiac magnetic resonance (ventricular ejection fraction and volumes) were also collected.

Patients included in this study are part of the cohort of PR patients recruited in a previous study (Rodríguez-Serrano et al., 2018) that has been submitted to pulmonary valve replacement (PVR) and clinically followed by one year. PVR was carried out on patients who met criteria according to the guidelines of clinical practice (Warnes et al., 2008; Baumgartner et al., 2010). A blood sample was taken for analysis of gene expression of β-ARs and GRKs in PBMC in this group of patients previous chirurgical intervention and at least one year after surgery. Clinical, electrocardiographic and RV assessment data were collected in the follow-up.

A group of healthy volunteers, comparable in age and sex with the patients, was included in the study to perform a single blood extraction for the analysis of gene expression of β-ARs and GRKs. Patients with inflammatory processes that could alter the expression of these molecules were excluded. The inclusion of control patients was performed in two different periods with a time lapse of 6–12 months between them and the results obtained were not statistically different (results not shown).

The study was made according to the Declaration of Helsinki and approved by the ethics committee of the Hospital La Fe with registry number 2011/0241.

### Analysis of Gene Expression of β-Adrenoceptors and GRKs in Peripheral Blood Mononuclear Cells

The biological material to study was a sample of fresh blood (10 ml) obtained by venipuncture and preserved in a tube with anticoagulant (EDTA). Immediately after the extraction, PBMC were isolated using Ficoll gradient (Montó et al., 2015). The sample obtained was stored at −80 °C until be processed.

Total RNA was obtained as previously described (Oliver et al., 2010). After RNA isolation, quality control was carried out by microfluidic electrophoresis using the Experion automated electrophoresis system (BioRad, Madrid, Spain) following the manufacturer’s conditions. Complementary DNA synthesis was carried out as previously described (Montó et al., 2015). The mRNAs encoding the human β-ARs (β1 and β2), the 3 GRKs mainly expressed in the myocardium (GRK2, GRK3, and GRK5), and glyceraldehyde-3-phosphate dehydrogenase (*GAPDH*) as internal standards were quantified by Taq-Man real-time RT polymerase chain reaction (PCR) with a GeneAmp 7500 Fast System (Applied Biosystems, Carlsbad, CA, United States). We analyzed (in duplicate reactions) a 10-fold dilution of the RT reaction of each sample using the TaqMan Gene Expression Assays (Applied Biosystems). The specific primer probes were: β1-AR, Hs00265096_s1; β2-AR, Hs00240532_s1; GRK2, Hs00176395_m1; GRK3, Hs00178266_m1; GRK5, Hs00178389_m1, and *GAPDH*, Hs99999905_m1 (Applied Biosystems). Real-time PCR reactions were done in 25 mL of TaqMan Universal PCR Master Mix (Applied Biosystems), including 5 mL of diluted RT reaction and 1.25 mL of 20 x TaqMan Gene Expression Assay Mix (250 nmol/L for the probe and 900 nmol/L for each primer). Complementary DNA was amplified following the manufacturer’ s conditions: one initial hold step at 95°C for 10 min, a second step with 40 cycles, 15 s at 95°C (denaturation), and 1 min at 60°C (annealing/extension). The targets and reference (*GAPDH*) were amplified in parallel reactions. The Ct values obtained for each gene were represented in the Supplementary Figure S1. We calculated the relative mRNA levels of β-ARs and GRKs referenced to *GAPDH* and converted into the linear form using the term 2^−dCt^ as a value directly proportional to the mRNA copy number (Montó et al., 2015).

### Statistical Analysis

The number of patients was adapted during the study, so in the Online Supplement the effects size with their corresponding confidence intervals are included. The statistical analysis was performed using Graph Pad software. A normality test, followed by one-way analysis of variance (ANOVA) and Student’s *t*-test or Newman Keuls’s test were used. Linear regression and Pearson’s correlation test were used to establish association between β-adrenoceptors and GRKs with hemodynamic and clinical variables in a post-hoc analysis. A probability value of *p* < 0.05 was considered significant.

## Results

### Study Population

23 patients with severe PR were included in the study (60.9% males, mean age of 35.7 ± 10.6 years). The most frequent underlying heart disease was Tetralogy of Fallot and pulmonary valve replacement with extension of the RV outflow tract was the most frequent surgical procedure. At recruitment, more than a half of patients were asymptomatic and 39.1% of the patients was under treatment with beta-blockers. The rest of the baseline characteristics of the population are shown in Table 1 and clinical variables in Table 2. According to the criteria of cardiac magnetic resonance, 47.8% had RV dysfunction (RV ejection fraction < 45%) and 73.9 % significant RV dilation (indexed RV end-diastolic volume measured by cardiac magnetic resonance ≥ 150 ml/m^2^). The comparisons were made with 22 healthy volunteers comparable in age and sex (62% males, mean age of 36.7 ± 5.2 years).

**Table 1:** Baseline characteristics of patients with severe pulmonary regurgitation.

Males, n (%)	14 (60.9)
Age (years)	35.7 +/− 10.6
Congenital heart disease, n (%)	
Tetralogy of Fallot	18 (78.3)
Valvular pulmonary stenosis	2 (8.7)
Other	3 (13.0)
Corrective intervention on the pulmonary valve, n (%)	21 (91.3)
Isolated miectomy /valvulotomy	5 (21.7)
Extension of the RV outflow tract	16 (69.3)
Functional class, n (%)	
NYHA I	15 (65.2)
NYHA II	8 (34.8)
Treatment with beta-blockers, n (%)	9 (39.1)
Bisoprolol	7 (30.4)
Atenolol	1 (4.3)
Carvedilol	1 (4.3)
Characteristics of ECG	
Sinus rhythm, n (%)	20 (87)
QRS duration (ms)	157.6 ± 26.3

*CMR: cardiac magnetic resonance; ECG: electrocardiogram; NYHA: New York Heart Association.*

**Table 2:** Clinical variables of patients with severe PR before and after PVR.

	Before PVR	After PVR
RVEDVi (ml/m2)	163.4 ± 24.1	101.7 ± 30.5^∗∗∗^
RVESVi (ml/m2)	87.7 ± 19.1	57.8 ± 20.4^∗∗∗^
RVEF (%)	44.4 ± 9.6	45.8 ± 9.4
LVEF (%)	59.4 ± 9.3	57.8 ± 10.2
NYHA (%)
I	65.2	91.3
II	34.8	8.7

*LVEF: ejection fraction of left ventricle, RVEDVi: right ventricular end-diastolic volume indexed, RVEF: ejection fraction of right ventricle; RVESVi: right ventricular end-systolic volume indexed. ^∗∗∗^ P < 0.001; Student t-test*

### Analysis of Gene Expression of β-ARs and GRKs in Peripheral Blood Mononuclear Cells From Patients With PR

**FIGURE 1:**
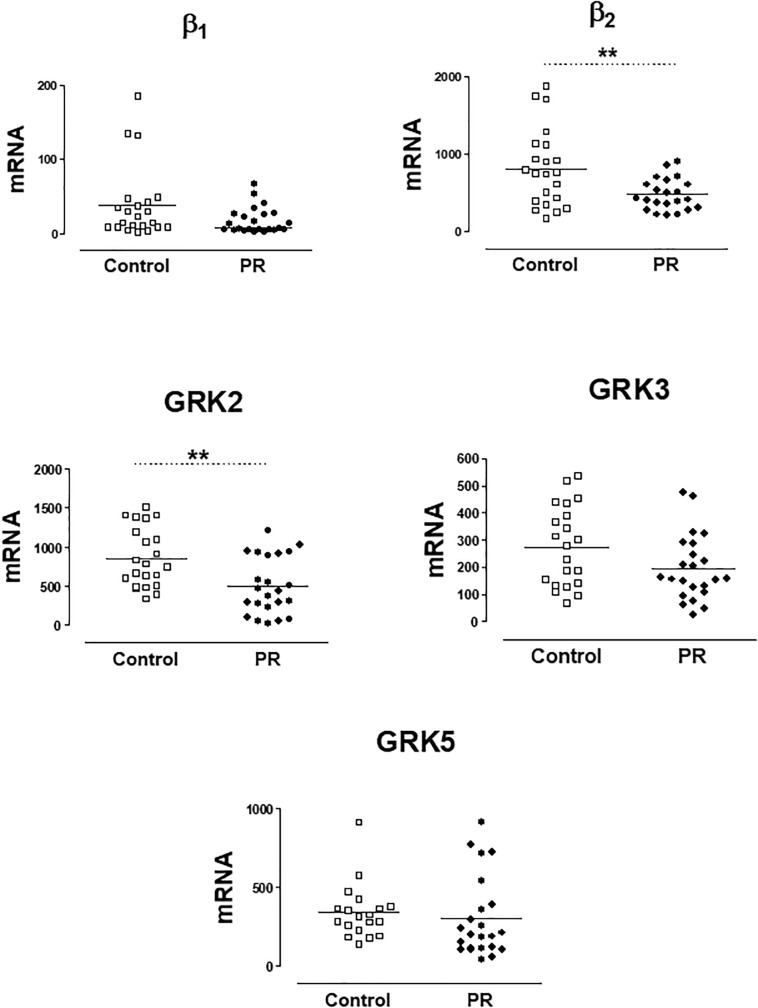
mRNA levels for β1- and β2-adrenoceptors, and G-protein coupled receptor kinases GRK2, GRK3, and GRK5, in peripheral blood mononuclear cells from healthy volunteers (control, *n* = 22) and patients with pulmonary regurgitation (PR, *n* = 23). Data were calculated as 2^-dCt^ vs. GAPDH as reference gene, and the mean was represented by the continuous line. ^∗^*P* < 0.05, ^∗∗^*P* < 0.01 (Student’s *t*-test).

In a previous work (Rodríguez-Serrano et al., 2018), we have found that patients with severe PR had lower genic expression of β2-AR and GRK2 in PBMCs. In the present study, the group of patients with severe PR also exhibit a significantly lower expression of β2-AR (PR, 513.8 ± 261.2 copies of mRNA) vs. healthy volunteers (control, 812.5 ± 497.2 copies of mRNA). The same occurs if we analyze GRK2 expression (503.3 ± 364.9 copies in PR vs. 858.1 ± 380.3 copies in control) The expression of β1-ARs, GRK3 and GRK5 did not change significantly in patients with severe PR vs. controls (Figure 1 and Supplementary Table S1).

**FIGURE 2:**
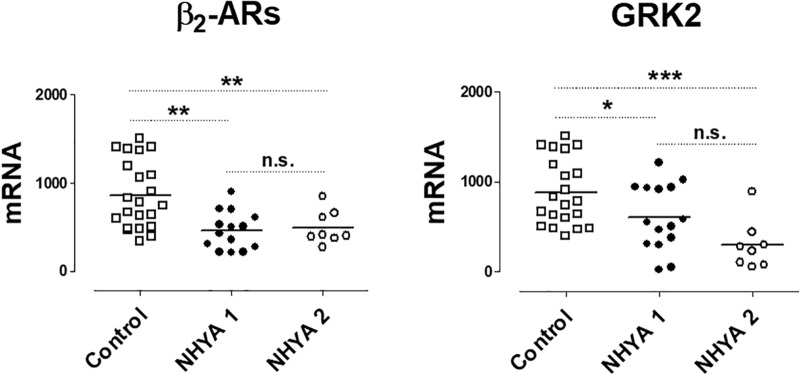
mRNA levels for β2-adrenoceptors (β2) and G-protein coupled receptor kinase 2 (GRK2) in peripheral blood mononuclear cells from healthy volunteers (control, *n* = 22) and symptomatic patients with pulmonary regurgitation, belonging to the NHYA class ≥ 2 (NHYA 2, *n* = 8) and asymptomatic patients, belonging to NHYA class 1 (*n* = 15). Data were calculated as 2^-dCt^ vs. GAPDH as reference gene and the mean was represented by the continuous line. ^∗^*P* < 0.05, ^∗∗^*P* < 0.01, n.s. = no significant (one way ANOVA and Newman Keuls’s test).

These results were also analyzed considering the NYHA functional class. Only the expression of GRK2 was lower in patients NYHA class ≥ 2 vs. patients in NYHA class 1 (Supplementary Table S2). When comparing the levels with the control group, as Figure 2 shows, the expression of β2-ARs and GRK2 significantly decreases as well as the NYHA class increases.

No differences were observed in gene expression of β-ARs and GRKs in PR patients in function of gender (results not shown), nor in patients under treatment with beta-blockers vs. non-treated patients (Table 3).

**Table 3:** mRNA levels for β-adrenoceptors and G-protein coupled receptor kinases GRK2, GRK3 and GRK5 in peripheral blood mononuclear cells from patients with pulmonary regurgitation under treatment (*n* = 9) or not (*n* = 14) with betablockers.

mRNA (2^−ΔCt^)	Beta-blockers	No beta-blockers	Effect size (confidence interval)
β1-adrenoceptor	20.23 (22.58)	16.12 (13.99)	−4.11 (−19.89 to 11.66)
β2-adrenoceptor	506.07 (350.40)	518.76 (199.67)	12.69 (−224.82 to 250.19)
GRK2	326.35 (287.40)	617.14 (372.74)	290.79 (−13.74 to 595.32)
GRK3	193.46 (128.13)	196.22 (121.07)	2.76 (−107.25 to 112.76)
GRK5	311.69 (258.1)	334.39 (263.59)	22.70 (−223.32 to 268.72)

*Values are expressed as 2^−dCt^ vs. GAPDH as reference gene and represented mean ± SD and the effect size with its corresponding confidence interval.*

**FIGURE 3:**
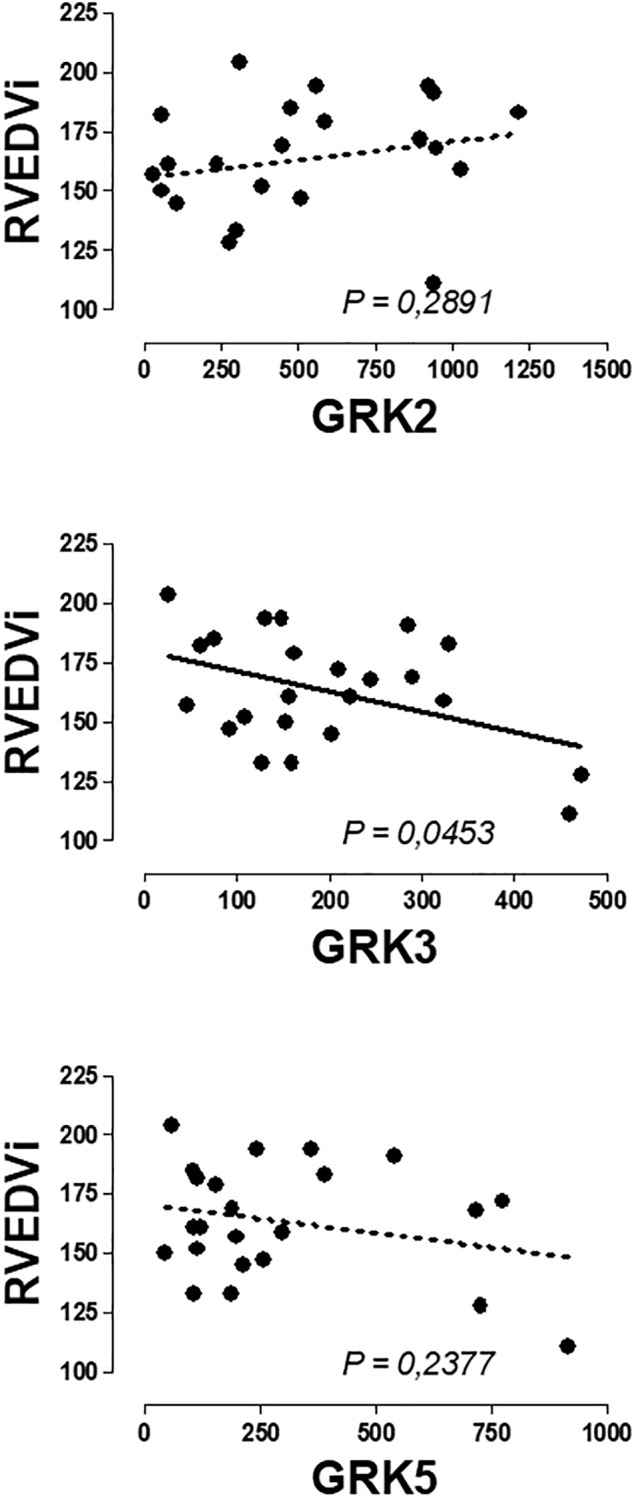
Graphical representation of the linear regression observed between the genic expression of GRK3 in the peripheral blood mononuclear cells of patients with pulmonary regurgitation. Significant linear regression and Pearson’s correlation if *P* < 0.05.

No significant correlations were found between the expression of β-ARs or GRKs with the age and the clinical variables of RV assessment. Only the expression of GRK3 was inversely related to the RVEDV (Figure 3 and Supplementary Table S3).

### Changes in Clinical Variables and Genic Expression of β-ARs and GRKs in PR Patients After PVR

The changes observed in the clinical, electrocardiographic and RV assessment after the PVR are shown in Table 2. Significant improvement was observed in the NYHA functional class of patients and RV volumes, indicating an adequate response after surgical intervention. All patients receiving beta-blockers before PVR continue with the treatment and two patients begin treatment after the surgery.

**FIGURE 4:**
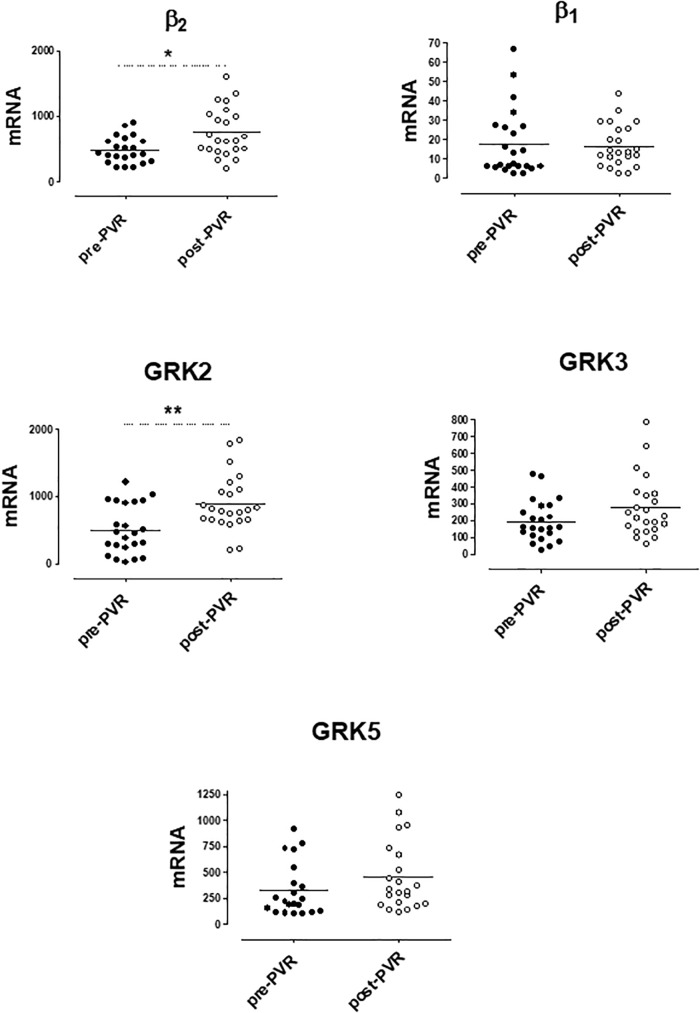
mRNA levels for β1- and β2-adrenoceptors and G-protein coupled receptor kinases (GRK2, GRK3, and GRK5) in peripheral blood mononuclear cells from patients with pulmonary regurgitation (*n* = 23) before (pre PVR) and after (post PVR) pulmonary valve replacement. Data were calculated as 2^−dCt^ vs. GAPDH as reference gene and the mean was represented by the continuous line. ^∗^*P* < 0.05; ^∗∗^*P* < 0.01 (Paired Student’s *t*-test).

mRNA levels of β-ARs and GRKs in PBMC after the PVR are shown in Figure 4. The lower expression of GRK2 and β2-AR observed in PBMC from PR patients respect to controls significantly increases after PVR (Figure 4 and Supplementary Table S4). No significant differences were found in the mRNA levels of β1-AR, GRK3 and GRK5 between PR patients before and after PVR (Figure 4 and Supplementary Table S4).

Supplementary Figure S2 show the analysis of results obtained before and after PVR for each patient included in the study. It is interesting that the increase in the expression of GRK2 and β2-ARs observed after PVR, implies the recovery of normal values not statistically different from those observed in the group of healthy subjects (Table 4).

**Table 4:** Comparison of the mRNA levels of β-adrenoceptors and GRKs in PBMC from healthy volunteers (control) and patients with pulmonary regurgitation after pulmonary valve replacement (PVR)

mRNA	Control	PVR	Effect size (confidence interval)
β1-adrenoceptor	30.9 ± 37.0	16.8 ± 11.1	14.07 (−2.2 to 30.4)
β2-adrenoceptor	812.5 ± 497.2	754.8 ± 372.7	57.8 (−205.7 to 321.3)
GRK2	858.1 ± 380.3	897.8 ± 419.1	39.6 (−280.7 to 201.4)
GRK3	292.4 ± 164.2	278.8 ± 181.0	13.63 (−90.5 to 117.7)
GRK5	342.0 ± 175.5	454.6 ± 333.2	−112.7 (−285.0 to 59.7)

*Values are expressed as 2^−dCt^ vs. GAPDH as reference gene and represented mean ± SD and effect size with its corresponding confidence interval*

### Significant Correlation Between mRNA Levels of b2-ARs and GRK2 in PBMC From Healthy Volunteers and Patients With Pulmonary Regurgitation

A linear regression analysis was performed between the mRNA values of β1-AR and β2-AR and the three GRKs isoforms expressed in PBMC obtained from healthy subjects (control group) and patients with pulmonary regurgitation (Supplementary Table S5).

**FIGURE 5:**
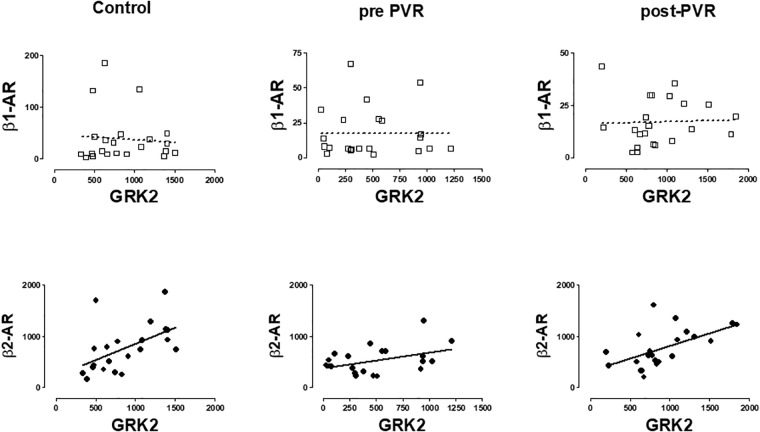
Graphical representation of the linear regression observed between the mRNA levels of β-AR subtypes (β1, β2) and GRK2 in PBMC from healthy volunteers (control) and patients with pulmonary regurgitation before (pre-PVR) and one year after (post-PVR) pulmonary valve replacement. Significant linear regression and Pearson’s correlation (*P* < 0.05) is represented as a continuous line and no significant regression (*P* > 0.05) as a discontinuous line.

A significant correlation was observed between β2-AR and GRK2 expression in healthy volunteers and in patients with PR. The same correlation was observed in this subgroup of patients after the chirurgical intervention. No correlation was observed between β1-ARs and GRK2 in any group studied. Figure 5 provides data on the relationship of mRNA expression of β1-ARs or β2-ARs and GRK2 in each subgroup of subjects.

Supplementary Table S5 includes a more complete description of the correlations obtained for the other GRKs isoforms but the more consistent observation is related to that described in Figure 5 and suggest the existence of a common regulatory mechanism for both genes as previously proposed (Montó et al., 2015).

## Discussion

Pulmonary regurgitation (PR) is a frequent and important consequence of the surgery performed in patients with Tetralogy of Fallot and pulmonary stenosis. Right ventricle is subject to chronic volume overload due to the presence of PR and it leads to its dilatation and dysfunction, associating arrhythmias, exercise intolerance, HF and death. PVR is indicated in symptomatic patients but this indication is still under debate in asymptomatic patients. Therefore, new parameters that identify the evolution of patients are being studied (Geva et al., 2018). At the time, as shown in the guidelines (Warnes et al., 2008; Baumgartner et al., 2010), assessment data of the right ventricle, such as end-diastolic volume, have been important for the PVR indication, but currently other data such as patient age, end-systolic volume and RV function seem more important (Frigiola et al., 2008; Geva et al., 2018). To expand the knowledge of this pathology, and as a useful tool to decide clinical interventions, it could be interesting to obtain a molecular marker that would orientate about the adequate time to PVR.

β1- and β2-ARs are expressed in PBMC, as well as three GRK isoforms: GRK2, GRK3 and GRK5 (Agüero et al., 2009; Oliver et al., 2010; Montó et al., 2015). The low mRNA level of β1-AR implies a poor protein expression and function of this subtype, being the β2-AR the main responsible of the adrenergic modulation of PBMC (Sanders, 2012). Alterations in the expression of β-adrenoceptors and GRKs have been observed in PBMC of patients with cardiovascular diseases such as hypertension or HF (Iaccarino et al., 2005; Penela et al., 2006; Vinge et al., 2007; Agüero et al., 2008, 2012; Oliver et al., 2010; Rengo et al., 2011; Santulli et al., 2011; Montó et al., 2015; Sato et al., 2015), and, in particular, GRK2 has been proposed as a biomarker for the evolution of the HF (Rengo et al., 2016). These alterations vary according to the origin (Montó et al., 2012), stage of the disease (Agüero et al., 2012), or use of betablockers (Leineweber et al., 2005). There is previous evidence that some changes in the heart are reproduced in PBMC (Brodde et al., 1986; Iaccarino et al., 2005; Oyama et al., 2005), and this would allow the use of PBMC as a mirror of cardiac changes. However, there is not always a concordance in the changes produced in heart and PBMC. So, high levels of GRK2 (Penela et al., 2006; Montó et al., 2015) and β2-AR (Montó et al., 2015) in myocardium of patients with dilated cardiomyopathy have been described, but our previous results indicate that the expression of GRK2 and β2-ARs decreases in PBMC of the same patients (Montó et al., 2015). However, the utility as biomarkers of β2-AR and/or GRK2 expression in PBMC could be the same if a direct relationship to pathology was established.

We have shown that β2-AR and GRK2 expression decrease in PBMC from patients with severe PR vs. healthy volunteers, as occurs in HF patients (Rodríguez-Serrano et al., 2018). Therefore, the expression of both genes in PBMC could be an interesting marker of the evolution of the PR patients. For this reason, in the present study, we analyze the mRNA levels of the β-adrenoceptors (β1 and β2), and the three GRKs (GRK2, GRK3, and GRK5) present in PBMC from symptomatic (NHYA ≥ 2 class) and asymptomatic patients (NHYA 1 class) with PR, before and after PVR, in order to determine its utility as clinical biomarkers.

Of the five genes studied, a markedly lower expression of GRK2, and β2-ARs in PBMC was found in the group of patients with PR vs. controls, as we described previously (Rodríguez-Serrano et al., 2018), together to a slight decrease in the GRK3 expression. However, the more interesting contribution of the present work is that the reduced expression of GRK2 and β2-ARs was found not only in symptomatic patients (NHYA ≥ 2 class) but also in asymptomatic patients (NHYA 1 class). Then, the decrease in the mRNA levels of β2-AR or GRK2 in PBMC could precede the clinical symptomatology and could alert about a deteriorated situation that other clinical parameters does not evidence. This could mean that, although the parameters used for the surgical indication are RV volume and function values, the points that are marked to indicate surgery may be late and the myocardial damage was already established before changes in these clinical variables.

Previous studies showed that betablockers up-regulate β-AR density in a β-AR subtype-selective manner, whereas they cause down-regulation of GRK2 (Iaccarino et al., 1998; Leineweber et al., 2005). However, this effect depends on the betablocker used, i.e., carvedilol exhibit a peculiar behavior decreasing β-AR density (Iaccarino et al., 1998). In our group of PR patients, a similar expression of β2-AR and GRK2 was observed independently of betablockers treatment, which excludes the possible bias due to the pharmacological treatment.

We performed an analysis of the possible correlation between PBMC genic expression of β-AR and GRKs and clinical variables characteristics of the pathology, but no significant correlation was observed between β2-AR and GRK2 expression and any of the variables studied, confirming that changes in the expression precedes and did not follow clinical symptoms. Therefore, the expression of these genes could be used as biomarker to detect cardiac damage even when this damage was not clinically very evident. An interesting result, not directly related to the main objective of the work, was the fact that a significant inverse correlation was found between GRK3 expressed in PBMCs and the right ventricular end diastolic volume indexed (RVEDVi) of patients with PR, adding more information to previous findings supporting a protective role of GRK3 expressed in human heart or PBMC in the cardiovascular system (Vinge et al., 2007; Oliver et al., 2010; Montó et al., 2012). Focus on the expression of GRK3 in heart and PBMC and its relationship to cardiac damage could be an interesting topic for future research and could contribute to a better understanding of the position of the GRK3 gene in a locus on a chromosome associated with left ventricular mass and contractility (Arnett et al., 2001).

At present, we do not know the mechanisms that leads to changes in the expression of β2-AR and GRK2 in PBMC from PR patients, and a similar decrease was found in PBMC from HF patients, which reverts after cardiac transplantation (Montó et al., 2015), confirming its relationship to the evolution of the pathology. For this reason, the objective of our study was to analyze the expression of β2-AR and GRK2 in PBMC of PR patients after PVR.

We observed significant differences in the expression of GRK2 and β2-AR in PBMC from PR patients before and after the PVR. In fact, PVR restored normal expression levels indicating a reversibility of the pathological situation involved in these changes.

It is interesting to note that a common pattern of changes between β2-AR and GRK2 genes has been previously described by us in different human and murine tissues (Montó et al., 2015) and the same occurs in PBMC from PR patients, where β2-AR and GRK2 expression decreases, whereas not significant changes were found in β1-AR or the other two GRKs studied (GRK3 and GRK5). In fact, genic expression of GRK2 and β2-AR in PBMC follow a common pattern of changes and a significant correlation was found between the mRNA levels of these genes in the different subgroups of patients studied (healthy volonteers and PR patients before and after PVR). This observation confirms the existence of a common regulatory mechanism.

As far as we know, our study is the first to analyze the behavior of β-ARs and GRKs in patients with congenital heart disease or with right ventricle disease in a stable situation. Only, one paper has been published analyzing alterations in the expression of GRK2 and ARs in atrial myocytes of patients with congenital heart disease operated on with cardiopulmonary bypass (Oliveira et al., 2017) but, in this case, the alterations were related to the vulnerability of the myocardium to catecholamine levels in an acute surgical context. These findings, although interesting, are not comparable to the situation of our patients, since post-RVP extraction has been performed in a stable situation, at least one year after surgery.

The decreased expression of β-ARs in PBMC has been related to a chronic inflammatory response (Grisanti et al., 2018). β2-AR expressed in immune cells are required for leukocyte recruitment to the heart following acute myocardial infarction. This response could be targeted to promote reparative processes while preventing chronic inflammatory events that are detrimental to healing (Grisanti et al., 2016).

Present results indicate that in PR patients, there is a lower expression of the β2-ARs and GRK2 expressed in PBMC. As we studied a mixed population of cells (PBMC) it is possible that the decrease in β2-AR and GRK2 in PR patients was general or limited to certain subsets of these cells and/or that altered composition of the PBMC population could be responsible for the low expression found, since the level of expression of β2-ARs is not similar among them (Murray et al., 1993). In any case, this change would be related to the right ventricle damage since it was similar to that found in PBMC from HF patients with damaged left ventricle (Rodríguez-Serrano et al., 2018). Therefore, the PBMC response to cardiac injury in PR patients mimics the response in patients with severe HF despite the PR patients are a population with few symptoms.

Analyzing the changes in the clinical variables after surgery, decrease of right ventricle volume and improvement of the functional class were observed, data similar to other studies (Ferraz Cavalvanti et al., 2013). The fact that gene expression of β2-AR and GRK2 in PBMC was normalized after PVR, as occurred in patients with HF after transplantation (Montó et al., 2015), indicates that a reversal of the response was obtained when cardiac damage disappears.

PR reappears after PVR in the follow-up, so new determinations when the dysfunction of the homograft or bio-prosthesis is detected could be useful for planning therapeutic measures. In some series analyzing long-term right ventricle remodeling (Hallbergson et al., 2015), data observed indicates that in the first two years after PVR there was a decrease in right ventricle volumes, but at 10 years after the chirurgical intervention, the volume of the right ventricle increased again to the values previous to the PVR, with a lower ejection fraction of the right ventricle, although there were no significant hemodynamic valve changes. This may indicate that ventricular remodeling, that has been caused by a chronic overload, makes the RV vulnerable and this can deteriorate again in the following.

A future multicentric study with prolonged follow-up after PVR intervention, including periodic measurements of GRK2 and/or β2-AR levels in PBMC, would be interesting to obtain prognostic data about the expression of GRK2 on PBMC as a biological marker to early detect myocardial damage in this population. At present, there are not clinical evidence that PVR improves prognostic in PR patients (Harrild et al., 2009; Ferraz Cavalvanti et al., 2013; Bokma et al., 2018). Furthermore, the protein expression has not been determined in the present work, due to the limited amount of samples. This limitation could be addressed in the future work by using flow cytometry to detect expression in the different PBMC populations.

## Limitations

The sample size of the study is small and so the statistical power limited, however, the sample is similar to that presented in previous publications on patients with PR (Ferraz Cavalvanti et al., 2013) in unicentric studies. Although the prevalence of patients with congenital heart disease is increasing (Baumgartner et al., 2010), it is still a pathology of low incidence compared to other heart diseases and therefore, there is a difficulty of carrying out more patient recruitment.

An important limitation of the article is that determination of protein levels of GRK2 and β2-AR has not been made. Post-transcriptional cellular events can modulate the translation of proteins, so it would have been interesting to see that changes in gene expression correlate with changes in protein levels, although available antibodies for β2-adrenoceptors are not enough reliable (Michel et al., 2009).

Gene expression of GRKs and β-adrenoceptors was analyzed only once after PVR. Long-term evolution of these molecular markers is unknown and they could change over time.

## Conclusion

Symptomatic and asymptomatic patients with PR exhibit decreased mRNA levels of β2-AR and GRK2 in PBMC that does not depend on the pharmacological treatment. This decrease is similar to that found in HF patients and is restored after PVR as occurs in HF patients after cardiac transplantation. This is the first study that evaluates these changes in patients with PR before and after PVR, and provides the first proof of concept about the use of these genes as potential biomarkers. Present findings support future studies with these molecular markers in order to analyze their prognostic value and usefulness to determine clinical interventions in these patients.

## Author Contributions

MR-S, JR, FB, LM-D, and PD contributed conception and design of the study. JR, AO, OC, and LM-D obtained clinical data. MR-S, FB, JA, and FM performed experiments. MR-S, FB, FM, JR, JA, AO, LM-D, and PD organized the database and analyzed data. MR-S, JA, and PD performed the statistical analysis. MR-S wrote the first draft of the manuscript. FB, FM, JR, and PD wrote sections of the manuscript. All authors contributed to manuscript revision, read, and approved the submitted version.

## Conflict of Interest Statement

The authors declare that the research was conducted in the absence of any commercial or financial relationships that could be construed as a potential conflict of interest.
