# Supplementary material for: β2-Adrenoceptors and GRK2 as Potential Biomarkers in Patients With Chronic Pulmonary Regurgitation
Source: Front Pharmacol. 2019 Feb 19;10:93. doi: 10.3389/fphar.2019.00093 (PMC6390728; doi:10.3389/fphar.2019.00093)
Supplement: Supplementary file 1 [file Table_1.docx]

**ONLINE SUPPLEMENT**

***Table S1. Comparison of the mRNA levels of β-adrenoceptors and GRKs in PBMC from healthy volunteers (control) and patients with pulmonary regurgitation***

| **mRNA** | **Control** | **Pulmonary Regurgitation** | **Effect size (confidence interval)** |
| --- | --- | --- | --- |
| **β1-adrenoceptor** | 37.9 ± 48.7 | 17.7 ± 17.5 | 20.2 (-1.7 to 42.0) |
| **β2-adrenoceptor** | 812.5 ± 497.2 | 513.8 ± 261.2 | 298.8 (55.9 to 541.8) |
| **GRK2** | 858.1 ± 380.3 | 503.3 ± 364.9 | 354.8 (130.7 to 578.8) |
| **GRK3** | 275.6 ± 32.2 | 195.1 ±121.0 | 80.4 (-1.370 to 162.2) |
| **GRK5** | 342.0 ± 175.5 | 301.8 ± 255.9 | 40.1 (-99.8 to 180.0) |

*Values are expressed as 2^-dCt^ vs GAPDH as reference gene and represented mean* ± *SD and effect size with its corresponding confidence interval*

***Table S2. Comparison of the mRNA levels of β-adrenoceptors and GRKs in PBMC from patients with pulmonary regurgitation according to NYHA class***

| **mRNA** | **NYHA class = 1** | **NYHA class**  ≥ 2 | **Effect size (confidence interval)** |
| --- | --- | --- | --- |
| **β1-adrenoceptor** | 15.9 ± 14.4 | 22.7 ± 22.4 | -7.6 (-23.5 to 8.3) |
| **β2-adrenoceptor** | 521.8 ± 297.7 | 498.7 ± 192.0 | 23.1 (-220.1 to 266.3) |
| **GRK2** | 612.2 ± 367.7 | 299.3 ± 274.3 | 312.8 (3.8 to 621.9) |
| **GRK3** | 174.7 ± 126.3 | 233.4 ± 107.4 | -58.7 (-168.2 to 50.9) |
| **GRK5** | 322.7 ± 248.1 | 331.9 ± 289.2 | -9.2 (-262.9 to 244.4) |

*Values are expressed as 2^-dCt^ vs GAPDH as reference gene and represented mean* ± *SD and effect size with its corresponding confidence interval*

***Table S3. Pearson’s correlation between mRNA levels of β-adrenoceptors and GRKs and the clinical variables determined in patients with pulmonary regurgitation***

|  | **RVEDVi** | **RVESVi** | **RVEF** | **Age** |
| --- | --- | --- | --- | --- |
| **β1-adrenoceptor** | r^2^ = 0.033  p = 0.882 | r^2^ = 0.007  p = 0.979 | r^2^ = -0.257  p = 0.236 | r^2^ = 0.125  p = 0.570 |
| **β2-adrenoceptor** | r^2^ = 0.198  p = 0.366 | r^2^ = 0.4  p = 0.112 | r^2^ = -0,072  p = 0.743 | r^2^ = 0.207  p = 0.343 |
| **GRK2** | r^2^ = 0.231  p = 0.289 | r^2^ = 0.199  p = 0.443 | r^2^ = -0.60  p = 0.787 | r^2^ = - 0.175  p = 0.424 |
| **GRK3** | r^2^ = -0.421  **p = 0.045** | r^2^ = -0.02  p = 0.994 | r^2^ = 0.026  p = 0.905 | r^2^ = 0.232  p = 0.288 |
| **GRK5** | r^2^ = -0.232  p = 0.312 | r^2^ = -0.006  p = 0.984 | r^2^ = -0.152  p = 0.512 | r^2^ = 0.131  p = 0.571 |

*RVEDVi = right ventricle end-diastolic volume indexed; RVESVi = right ventricle end-systolic volume indexed; RVEF = right ventricle ejection fraction*

*p< 0.05 indicates a significant correlation*

***Table S4. Comparison of adrenoceptors and GRKs between PR group before and after RVP with t de Student paired samples; expressed by mean, SD and the corresponding confidences intervals of the difference.***

| **mRNA (2^-ΔCt^)** | **PR before RVP** | **PR after RVP** | **Effects sizes** |
| --- | --- | --- | --- |
| **β1-adrenoceptor** | 17.73 (17.47) | 16.82 (11.13) | - 6.38 to 8.18 |
| **β2-adrenoceptor** | 513.79 (261.24) | 754.79 (372.67) | - 424.79 to - 57.21 |
| **GRK2** | 503.35 (364.95) | 897.76 (419.06) | - 631.38 to – 157.42 |
| **GRK3** | 195.14 (120.97) | 278.81 (181.01) | - 175.65 to 8.31 |
| **GRK5** | 325.75 (255.21) | 259.38 (149.06) | -47.08 to 179.81 |

***Table S5****.* ***Pearson’s correlation between mRNA levels of β-adrenoceptors and GRKs determined in PBMC from healthy volonteers (controls) and patients with pulmonary regurgitation before (pre-PVR) and after (post-PVR) pulmonary valve replacement (PVR).***

|  |  | GRK2 | GRK3 | GRK5 |
| --- | --- | --- | --- | --- |
| Control | B1 | 0.7261 | 0.4419 | **0.0021** |
|  | B2 | **0.0133** | **0.0230** | 0.1659 |
| Pre-PVR | B1 | 0.9876 | 0.8162 | 0.8602 |
|  | B2 | **0.0490** | 0.0867 | 0.0810 |
| Post-PVR | B1 | 0.8678 | 0.3272 | 0.4623 |
|  | B2 | **0.0057** | 0.1158 | 0.6289 |

*p< 0.05 indicates a significant correlation*

***Figure S1.*** *GAPDH, β2-AR and GRK2 expression in PBMC from healthy volunteers (control) and patients with pulmonary regurgitation before (pre-) and after (post-) pulmonary valve replacement (PVR). Data are expressed as 2^Ct^*

******

***Figure S2.*** *mRNA levels of β-adrenoceptors and GRKs determined in PBMC from patients with pulmonary regurgitation before (pre-PVR) and after (post-PVR) pulmonary valve replacement (PVR)*
